# Oxytocin receptor gene expression in the basal forebrain in autism: association with receptor binding levels and single nucleotide polymorphisms

**DOI:** 10.1186/s11689-026-09678-0

**Published:** 2026-02-19

**Authors:** Ethan E. Dayley, Susan Durham, Michelle C. Palumbo, Jill F. Lundell, Sara M. Freeman

**Affiliations:** 1 Department of Biology, Utah State University, Old Main Hill Logan, Logan, UT 84322 USA; 2 Ecology Center, Utah State University, Logan, UT USA; 3 Department of Behavioral Neuroscience, Oregon Health & Science University, Portland, OR USA; 4 MIT Lincoln Laboratory, Massachusetts Institute of Technology, Lexington, MA USA

**Keywords:** Autism, Oxytocin, Cholinergic neurons, Basal forebrain, Nucleus basalis, Ventral pallidum

## Abstract

**Background:**

The brain’s oxytocin system has been implicated in the neurobiology of autism (ASD), given the role of oxytocin in modulating social function in humans and animals more broadly. Previous work from members of our group reported dysregulation in oxytocin receptor (OXTR) binding in postmortem tissue from the basal forebrain in donors with autism compared to unaffected control donors. This study follows up on those findings by investigating the potential genetic and gene expression changes that could be driving those differences.

**Methods:**

We used adjacent sections from the same specimens from our previous study and performed duplex fluorescence in situ hybridization to visualize and quantify *OXTR* mRNA in the ventral pallidum (VP) and in the cholinergic magnocellular neurons of the nucleus basalis of Meynert (NBM), visualized with choline acetyltransferase (*ChAT*). We genotyped the brain samples using a SNP microarray on extracted DNA. We then used regression models to test associations between OXTR binding density, *OXTR* mRNA levels, and relevant *OXTR* SNPs. Additionally, we tested for correlations between age and *OXTR* mRNA.

**Results:**

ASD specimens showed significantly greater *OXTR* mRNA than unaffected donors in both the VP and the NBM. Furthermore, this is the first demonstration of *OXTR* expression in the cholinergic neurons of the human basal forebrain; 73% of *OXTR* signal in the images of the *ChAT*+ neurons were colocalized with the cholinergic neurons. OXTR binding levels from our previous study were positively associated with *OXTR* mRNA in the NBM but only in the *ChAT*+ neurons there. OXTR binding levels were not associated with *OXTR* mRNA in the VP. We genotyped all specimens for three common SNPs in the *OXTR* gene that have been associated with ASD in the literature, but none significantly predicted levels of OXTR binding or gene expression in the NBM or VP. OXTR mRNA levels were strongly positively correlated with donor age across regions, which was driven by ASD specimens.

**Conclusions:**

Taken together, our results contribute to a more nuanced picture triangulating variation in *OXTR* gene sequence, gene expression, protein levels, and human behavior.

**Trial registration:**

Clinical trial number: not applicable.

**Supplementary Information:**

The online version contains supplementary material available at 10.1186/s11689-026-09678-0.

## Background

Autism spectrum disorder (ASD) is a pervasive neurodevelopmental disability that is characterized by persistent differences in communication, sensory sensitivity, and rigid adherence to routine [1]. ASD prevalence estimates haven risen significantly over the past few decades, with current prevalence for children in the US estimated at about 1 in 36 [2]. This rise in estimated prevalence is controversial, with some studies suggesting that shifts in environmental factors might be driving a true shift in prevalence, while others suggest that new institutional changes in diagnostic criteria are simply driving an increase in awareness [3]. Regardless of the reason, the rise highlights the necessity of basic research into the etiology of ASD. Mental health struggles are unfortunately common in the autistic community and may contribute to low rates of both employment and independent living for autistic individuals [4, 5]. By developing our understanding of the biological processes that create differential outcomes and behaviors for autistic individuals, we may be better able to improve life outcomes for many individuals with autism.

The genetics of autism have been extensively studied, with multiple large-scale research projects giving insight into heritability and genetic associations. Autism is known to have a strong genetic component, with heritability estimates from large-scale studies ranging from 50 to 90% [6, 7]. Autism is also known to be polygenic and most likely involves interactions from both rare and common variants [8, 9]. Meta-analyses of autism genetics have revealed several significant risk alleles, including some associated with the oxytocin receptor gene (*OXTR*) [8, 10]. Because the oxytocin (OXT) system is known for its role in social interaction [11], which is one of the core symptoms of ASD, studying the oxytocin receptors (OXTR) of the brain is especially relevant to improving our understanding of the neurobiology of ASD.

Given its association with ASD, the OXT system has become a target for treatment of the social symptoms of ASD in many studies over the past two decades. Intranasal oxytocin (IN-OXT) administration has been a particularly active area of study, albeit with somewhat mixed results. Recent meta-analyses suggest that IN-OXT has beneficial effects on social functioning without apparent adverse effects [12, 13]. That said, the effects appear to be specific to social symptomology of ASD, with little to no effects on non-social domains. A potential factor that could have contributed to a lack of effectiveness of IN-OXT in some prior studies is whether or not IN-OXT administration was paired with behavioral therapy in a clinical context [14]. This idea is consistent with some more recent IN-OXT study results, although additional research is needed [15, 16]. It should also be noted that camouflaging or “masking” behaviors may complicate interpretation of behavioral studies since external behaviors might not correlate with internal state [17, 18]. However, fMRI studies have consistently shown differential brain network activation in autistic individuals following IN-OXT administration [19]. Affected regions included the amygdala, basal ganglia, frontal and prefrontal cortex, cingulate cortex, and parts of the occipital, temporal and parietal lobes [19]. Although it is still unclear to what degree IN-OXT administration affects OXT levels in the cerebrospinal fluid [20, 21], the studies above provide strong evidence for the involvement of the OXT system in ASD.

The strong evidence of a link between ASD and OXT highlights the importance of direct brain studies to contextualize IN-OXT studies and better understand what neural circuits may be involved. A 2018 study from members of our group examined OXTR binding density in the basal forebrain and midbrain and found significant differences between autistic and allistic (AST; non-clinical, unaffected) individuals within two brain regions: the nucleus basalis of Meynert (NBM) and ventral pallidum (VP), which are regions associated with visual attention and the mesolimbic reward pathway, respectively [22]. In the NBM, OXTR density was significantly increased in autistic donors, whereas in the VP, OXTR density was significantly decreased in ASD compared to AST. These results suggest that autistic individuals are differentially sensitive to OXT within those regions, which could have behavioral implications, although it is speculative at this point. For instance, since the VP is part of the mesolimbic reward pathway, a reduced ability of OXT to activate OXTR in that area may contribute to a reduced experience of social reward or social motivation in autism. The NBM is a cholinergic region of the basal forebrain that has been implicated in selective and sustained visual attention and has projections to multiple other brain regions, including the amygdala and cortex [23], both of which show altered activation following IN-OXT administration [19]. Increased OXTR density (and thus sensitivity to OXT) of the NBM could contribute to the differences in social attention that have been reported in ASD [24–26].

To follow up on these results, the current study seeks to examine whether *OXTR* gene expression shows the same pattern of differential levels in ASD compared to AST as its protein product. We used adjacent sections from the same postmortem specimens as the 2018 study to visualize and quantify *OXTR* mRNA transcripts using fluorescence in situ hybridization. This approach is unique because most studies of *OXTR* expression in the human brain have relied on a single open-source transcriptomic data set from six allistic donors [27, 28], and to our knowledge, the studies to date quantifying *OXTR* expression in postmortem brain tissues from donors with ASD have used qPCR on tissue homogenates [29, 30]. Both of these approaches sacrifice cellular neuroanatomy, which we have preserved in the current study by working with tissue sections. We hypothesized that *OXTR* mRNA expression in the NBM and VP would align with our receptor binding results and show the same group differences that we previously described between ASD and AST. We also analyzed DNA from all specimens for three *OXTR* single nucleotide polymorphisms (SNPs) implicated in risk for ASD (rs2268491, rs2268495, rs237885) in an effort to link genetic variation with gene expression and protein density.

## Materials and methods

### Specimens and tissue preparation

A total of 44 frozen blocks of de-identified, unfixed, frozen postmortem human brain tissue from the basal forebrain containing the NBM and/or VP were previously provided by the University of Maryland Brain and Tissue Bank, a brain and tissue repository of the NIH NeuroBioBank. Out of these samples we selected a total of 17 ASD and 24 AST specimens (41 total) for analysis based on presence of identifiable NBM or VP regions in the 2018 study. These specimens were age-, sex-, and race-matched across AST and ASD groups to the best of our ability, with recommendations and approval from the staff of the NeuroBioBank (as described in our previous study). The specimens were stored at −80°C and had previously been brought to −20°C for cryosectioning at 20 µm. Sections were mounted to Fisher Superfrost-Plus slides, sealed in a slide box with a desiccant packet, and returned to −80°C storage until use in fluorescence in situ hybridization.

### Fluorescence in situ hybridization (fISH) assay

To visualize *OXTR* mRNA within the NBM and VP we used the RNAScope® Multiplex Fluorescent v2 Assay [31] according to the manufacturer’s instructions (Advanced Cell Diagnostics, Inc) for unfixed, frozen brain tissue, with some modifications. Sealed slide boxes were thawed for 1 h at room temperature (RT) before opening. Sections were fixed overnight in 4% paraformaldehyde (pH 7.4) at 4°C. After washes and dehydration in ethanol, endogenous peroxidases were quenched with 0.3% H_2_O_2_ for 10 min at RT. Slides were then boiled for 10 min in the Target Retrieval Reagent, rinsed in ddH_2_O, then incubated for 30 min at RT in RNAScope Protease IV before probe hybridization. We used pooled multiplex probes containing a probe for the human *OXTR* gene and a probe for the human choline acetyltransferase gene (*ChAT*), to colocalize *OXTR* in the cholinergic neurons of the basal forebrain. This approach allowed us to specifically quantify *OXTR* expression in the cholinergic neurons of the NBM as well as in the surrounding basal forebrain and VP. Positive control probes (targeting housekeeping genes *PPIB* and *Polr2a* genes) and negative control probes (targeting bacterial gene *dapB*) were used on adjacent sections from every specimen. Probes were amplified according to manufacturer’s instructions then visualized with distinct Opal dyes that do not overlap in their emission spectra: Opal 570 and 690 (equivalent to Cy3 and Cy5.5, respectively). *OXTR* was visualized with Opal 570 (red) and *ChAT* was visualized with Opal 690 (far-red, or pink). Through optimization experiments using postmortem human brain tissues, we have learned to avoid the use of any green fluorescent dyes (GFP/Opal 520) due to autofluorescence in this spectral range from fixatives or lipofuscin, which accumulates in aged human tissues [32]. Prior to coverslipping, cell nuclei were stained with DAPI, a standard fluorescent marker of cell nuclei. All slides were kept in the dark at °C after coverslipping to minimize fluorescent signal loss before quantitative imaging. During the assay, two samples had their pooled experimental probes accidentally mixed with negative or positive control probes and had to be excluded from the study. Final sample sizes for our two groups were: 17 ASD and 22 AST.

### Imaging

All slides were stored in the dark at °C for an average of 30 days (range of 20–52) prior to imaging. A fluorescence microscope (BZ-X810, Keyence, Itaska IL, USA) was used to image all slides. For all images, we set magnification at 20× with 40% excitation light and standard resolution. Identical exposure and excitation settings were used across all sections; exposure times were set to 1/5 s for DAPI, 1/4 s for *OXTR*, and 3 s for *ChAT*. Sections receiving the positive and negative control probes were used to optimize these exposure settings to prevent over exposure of abundant signals and minimize non-specific background. Example images from our positive and negative controls are included in Supplementary Fig. 1. Because the red and far-red coloration scheme of our selected Opal dyes were difficult to distinguish visually, we chose to change the microscope’s pseudocoloration settings for each channel so that *OXTR* mRNA appears green and *ChAT* mRNA appears red. For quantification, our target number of images per specimen was four per region of interest, and we captured two to five images each from the NBM and VP for each specimen, depending on the size of the region. We also captured images showing the neuroanatomical location of each quantifiable image based on the DAPI, *ChAT*, and *OXTR* signals for later quality control and traceability. We additionally captured a larger image of each sample at 4 × magnification for most specimens and compared these to the acetylcholinesterase counterstained images and OXTR film autoradiograms from the original study to confirm our selected neuroanatomical locations for mRNA quantification and ensure that they were taken in the corresponding region to where OXTR binding had been quantified for NBM and VP in our 2018 study. For a small number of representative samples, we also captured a select few 60X images targeting the *ChAT*+ neurons to better demonstrate the *OXTR* signal there.

### Quantification

Images were checked for quality and were excluded for one of two reasons. The first reason was non-matching histology. For the NBM, image inclusion required the presence of cholinergic magnocellular neurons (identifiable by the presence of condensed *ChAT* signal surrounding cell nuclei). For the VP, image inclusion required being located below the anterior commissure according to brain atlas images, appearing as homogeneous tissue without white matter tracts or other significant intrusions, and the presence of *OXTR* mRNA and absence of significant *ChAT* mRNA signal. Two samples for the NBM and two for the VP were dropped from the analysis due to aberrant or unidentifiable histology. The second potential reason for exclusion was incorrect imaging parameters. These were verified both by visual inspection and by checking image metadata. One VP sample was excluded due to incorrect imaging parameters. Two more samples were also accidentally lost. Our final sample sizes were 30 NBM samples (12 ASD and 18 AST), and 21 VP samples (7 ASD and 14 AST). Of these, 26 NBM (11 ASD and 15 AST) and 18 VP (6 ASD and 12 AST) samples had corresponding autoradiography data from the previous study and comprised our final full dataset.

Because our study was conducted across three assays (due to limitations in the number of slides that can be processed together in a single assay), we also checked for batch-wise differences across assays that could have influenced our results. We used an ANOVA to compare brightness for each channel across assays and found no significant differences (Supplemental Materials).

**Fig. 1:**
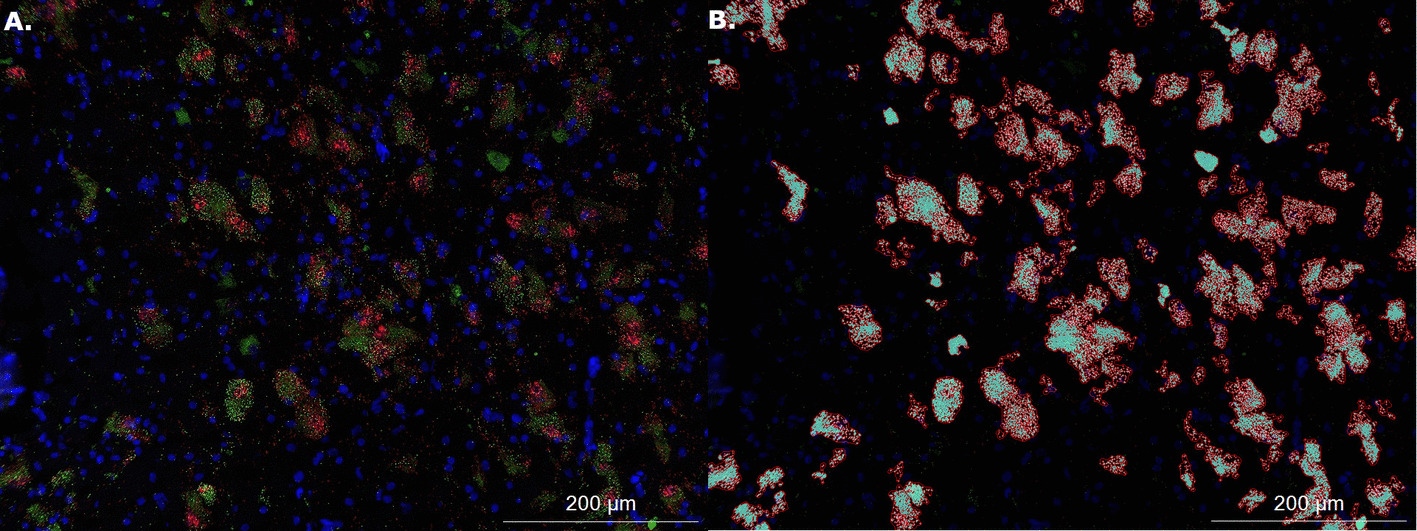
Thresholding image extraction process for quantification. Panel **A** shows a 20X image with *OXTR* mRNA signal in green and *ChAT* mRNA signal in red. Panel **B** shows the results of the thresholding process to isolate only *OXTR* signal within the boundaries of the *ChAT* + areas

The Hybrid Cell Count function in Keyence BZ-Analyzer software was used to quantify signal brightness and area from all images. This software uses a channel-by-channel thresholding algorithm to extract fluorescence signal intensity (brightness) and signal area (µm^2^). We ran our analyses on both signal intensity (brightness) and signal area and report as our primary outcome the results from signal area as a metric of gene expression (Note: signal brightness recapitulated all findings and the entire set of those results are included in Supplementary Materials). Each thresholded mask was made by manually adjusting the sensitivity and tolerance of the mask parameters on one representative image until the pattern of quantified pixel area aligns with true pattern of that channel’s signal on the image being quantified. We then saved those settings as a macro and applied it to all images in order to standardize the quantification approach. All of the resulting masks generated by the saved macros were visually checked to ensure that the quantified pixel area aligned with the visual distribution of signal for every channel for every specimen. For the VP, we extracted mean signal brightness and area from all 20× images for the *OXTR* channel; we refer to this metric as VP *OXTR*. Since the NBM is defined by its composition of large clusters of cholinergic magnocellular neurons, we used them as a marker for where to image. However, the original study quantified macroscopic differences within the NBM as a whole, because autoradiography is not a technique that provides cellular resolution. So, we took three measures of NBM *OXTR* mRNA signal to ensure we did not miss potential sources of variation. First, we quantified the *OXTR* signal in the NBM in the same way we quantified it in the VP: across the entire 20 × image without taking into account the locations of cholinergic neurons; we refer to this metric as NBM *OXTR* area. We also calculated the colocalization of *OXTR* expression with regions of condensed cholinergic signal. To do so, we used the software’s single extraction function to designate a target area for *OXTR* extraction using the *ChAT* channel signal. We then filled cracks to make the cholinergic regions continuous. Next, we extracted the *OXTR* channel area from within those cholinergic areas. Once we extracted the *OXTR* area values, we divided each by the area of cholinergic signal for that image to account for differences in the number and size of cholinergic neurons across images. For a representative example of the *OXTR* extraction process within condensed cholinergic regions see Fig. 1. We named this metric NBM *ChAT*+ *OXTR* area. We also took an inverse metric which only examined *OXTR* mRNA signal outside condensed cholinergic regions and named it NBM *ChAT- OXTR* area. Percent colocalization to *ChAT* of the total *OXTR* signal was calculated as the ratio of *ChAT*+ *OXTR* to total *OXTR* area in the NBM, summing values from each image on a sample-wise basis prior to calculating mean and standard error (across all samples and stratified by neurotype).

### *OXTR* SNP analysis

We extracted DNA from 42 of our original 44 brain samples using a Qiagen kit (2 specimens were dropped due to tissue integrity issues resulting in unrecognizable anatomy, preventing us from being able to associate SNPs with OXTR neural phenotypes in those samples), and the purity and concentration of all resulting DNA samples were confirmed using a fragment analyzer. The concentrations of samples were normalized to 50 ng/µL, and 10 µL of each sample was sent to the Genome Core at the University of California Davis for SNP analysis. We used two, 24-sample Illumina Infinium Global Screening Array (GSA) v3.0 BeadChips. The GSA included over 630,000 known SNPs across the human genome. Of the total SNPs genotyped in our samples, only three in the *OXTR* gene had previously been reported to be associated with an increased risk of ASD: rs2268491, rs2266495, and rs237885 [10]. A formal evaluation of our full genome-wide SNP dataset is ongoing; the current study focused only on those three *OXTR* SNPs to assess whether genotype for these loci was associated with *OXTR* gene expression levels or OXTR binding density in our samples.

### Statistical analysis

We regressed each *OXTR* mRNA metric on neurotype (ASD or AST). We checked for normality and homoskedasticity of residuals using the ‘simulateResiduals’ function from the *DHARMa* 0.4.6 R package [33] and found that two of the metrics (*ChAT* + *OXTR* area and *ChAT- OXTR* area) were normally distributed, whereas NBM *OXTR* area and VP *OXTR* area both required a square root transform. We then calculated p-values for our linear models and calculated estimated marginal means, standard errors, and 95% confidence intervals based on our models using the *emmeans* R package [34]. To evaluate whether OXTR protein levels were correlated with *OXTR* gene expression, we calculated correlation coefficients between OXTR binding density from the original study and our four *OXTR* mRNA metrics.

To analyze whether any of our three target SNPs were significantly associated with OXTR binding density or gene expression across our samples, we first checked for linkage disequilibrium between our SNPs using the LDMatrix tool to obtain D' values for each SNP pairing [35]. Since our D' values were medium to high [0.328–0.625], we opted for ridge (L2-penalized) regression to deal with the multicollinearity issues which linkage disequilibrium can cause. To check for effects by neurotype, we generated separate models for ASD and AST neurotypes, as well as combined models, for both the NBM and VP. For each model, we used *cv.glmnet* to select λ (a tuning parameter which determines the amount of shrinkage) with five-fold cross-validation with the λ which minimized the cross-validation prediction error rate to create an L2-penalized model using *glmnet* [36, 37]. We used the fitted model to generate R^2^ values and obtained p-values for each beta estimate using permutation tests with 1000 permutations. We corrected the p-values for multiple comparisons using the FDR method [38].

To follow up on age-related findings in our prior study, we tested for correlations between age and *OXTR* mRNA levels using Pearson’s correlations, which were calculated in two ways in both regions of interest: across all subjects and in separate groups by neurotype.

## Results

### Anatomical characterization of *OXTR* gene expression in the human basal forebrain

**Fig. 2:**
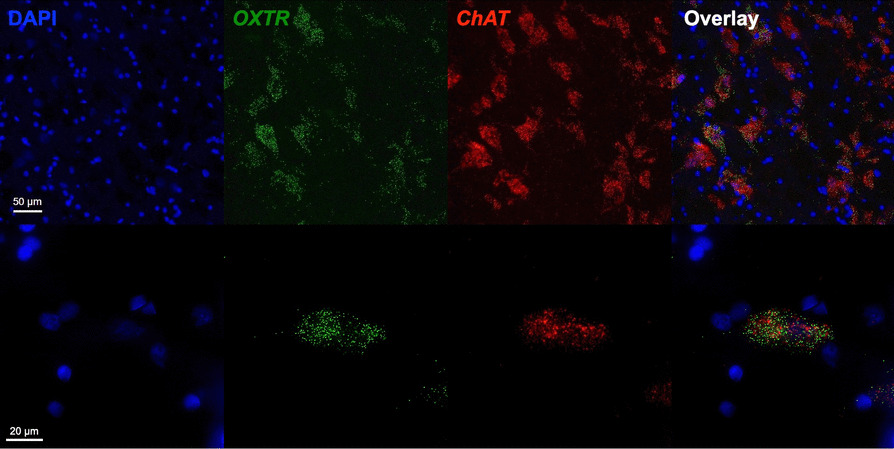
Fluorescence images across channels for multiplex *OXTR* and *ChAT* in the human basal forebrain. Top row: Representative 20X images from across all three imaging channels, plus overlay, showing the high degree of overlap in *OXTR* and *ChAT* gene expression. Scale bar = 50 µm. Bottom row: Representative 60X images from across all three imaging channels, plus overlay, showing the high degree of overlap in *OXTR* and *ChAT* gene expression. Scale bar = 20 µm

**Fig. 3:**
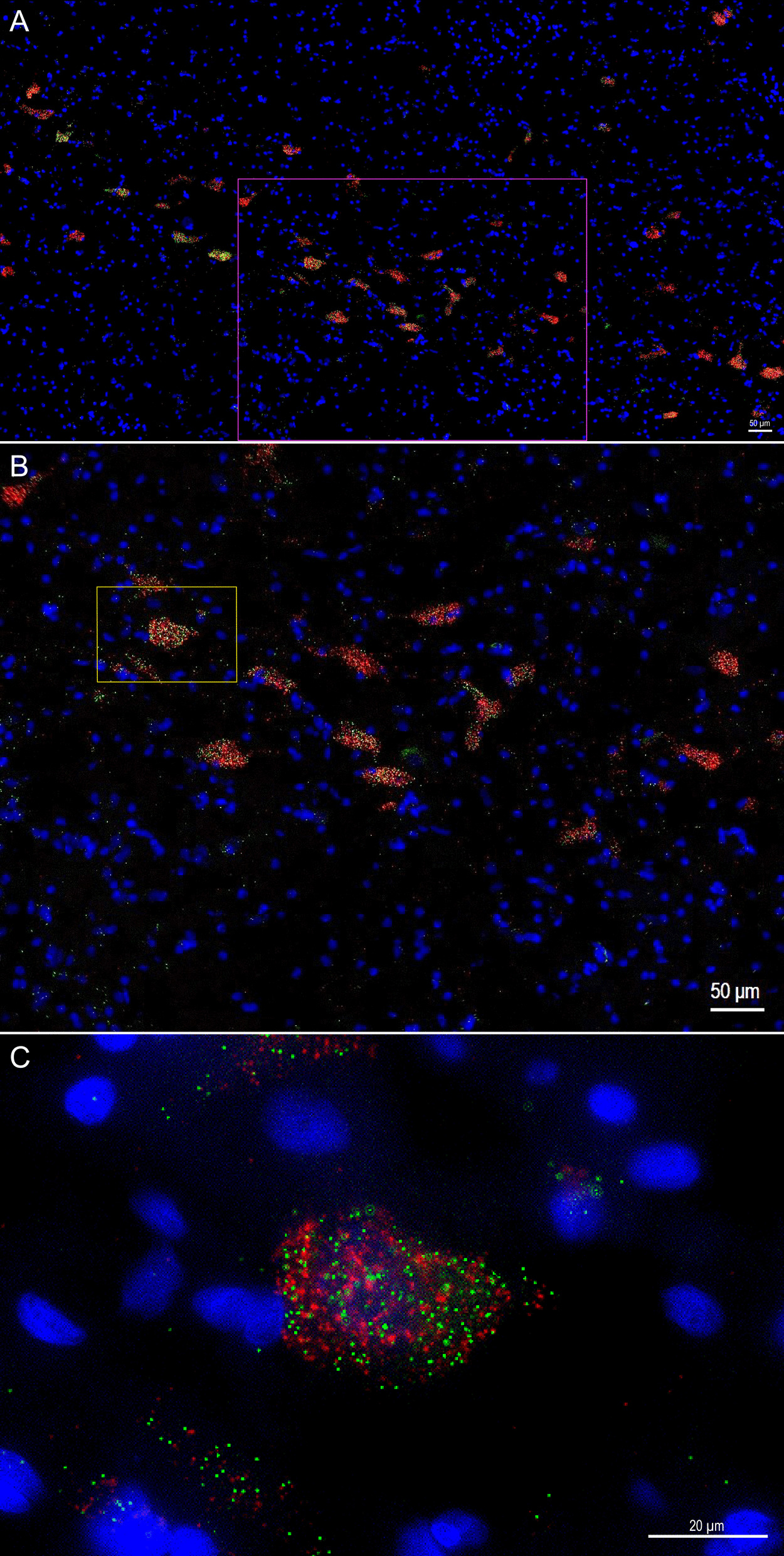
Colocalization of *OXTR* mRNA with the cholinergic neurons of the human basal forebrain. **A** Overview image of a band of *ChAT*+ neurons (red) containing *OXTR* mRNA (green), with the pink box indicating the zoomed in 20X image shown in panel B. **B** 20X image showing *OXTR* location across these *ChAT*+ neurons, with the yellow box indicating the location of the 60X image shown in panel C. **C** 60X image showing the striking colocalization of *OXTR* mRNA within the *ChAT*+ area. Scale bar in A-B is 50 µm; scale bar in C is 20 µm

We report the first anatomical colocalization of *OXTR* mRNA in the *ChAT*+ cholinergic magnocellular neurons of the human basal forebrain (Figs. 2 and 3). While we also found widespread *OXTR* mRNA throughout the VP, which has interesting implications for future studies of the dopaminergic neurons in this area, our most notable results in the anatomical characterization of *OXTR* gene expression in the human basal forebrain are the striking colocalization patterns between *OXTR* and *ChAT* mRNA. Our use of a *ChAT* probe for duplex fluorescence ISH with *OXTR* was primarily intended to anatomically identify the cholinergic neurons that comprise the NBM, but it quickly became apparent that the high degree of overlap between these two gene expression patterns was a primary outcome of our study (Fig. 3). We found that 73 ± 3.8% of the *OXTR* signal in the NBM images was colocalized with the *ChAT* signal. Stratified by neurotype, the percent colocalization for *ChAT*+ and *OXTR*+ areas was 74.2 ± 5.7% for ASD, and 72.2 ± 5.3% for AST, which are not statistically different.

### *OXTR* mRNA comparison between neurotypes

**Fig. 4:**
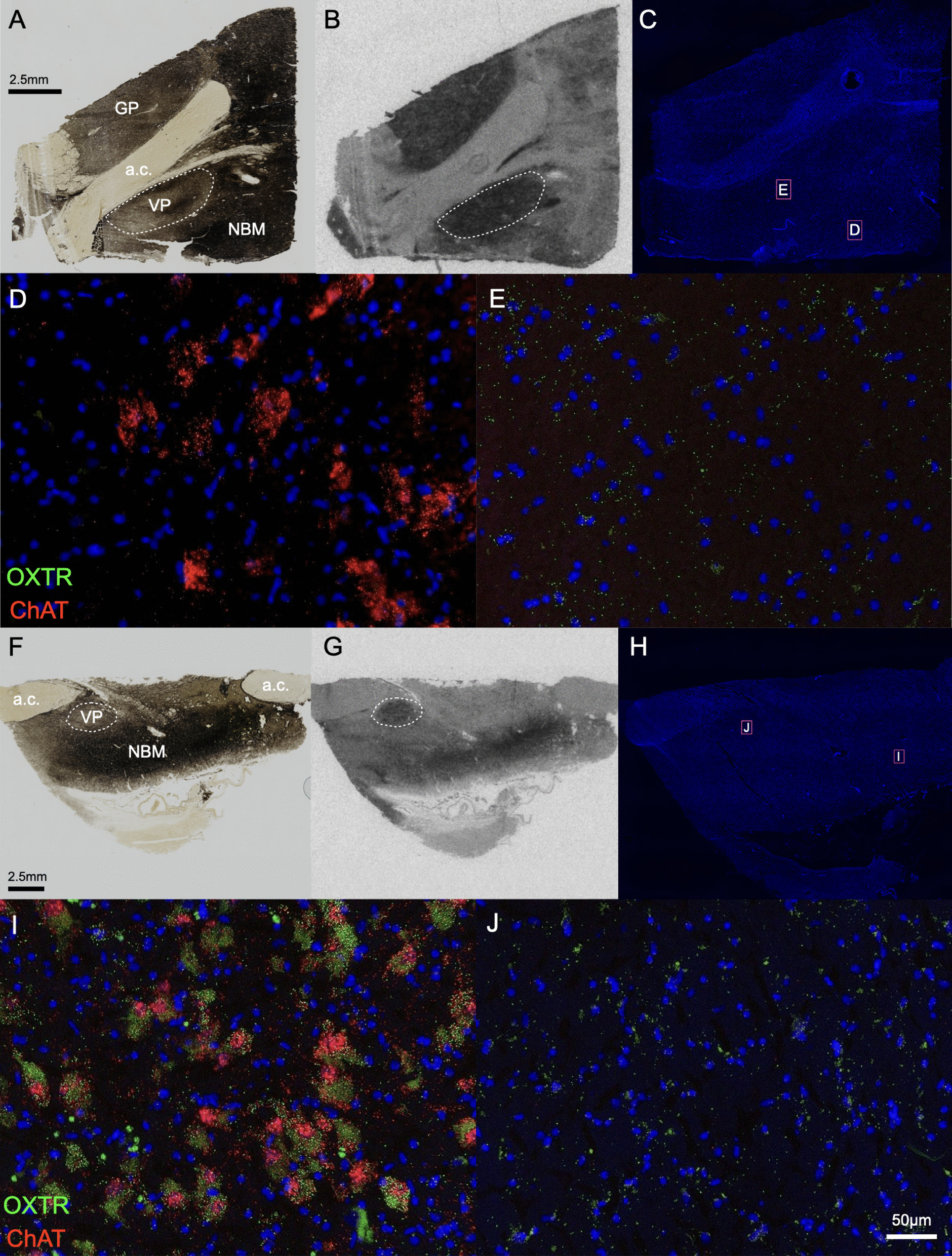
Representative basal forebrain images in each neurotype. Example allistic specimen (**A**-**E**) and autistic specimen (**F**-**J**). **A**, **F** Acetylcholinesterase stained section, which delineates the heavily cholinergic nucleus basalis of Meynert (NBM) and adjacent ventral pallidum (VP), globus pallidus (GP), and anterior commissure (a.c.). **B**, **G** Film autoradiograms for the sections in **A**, **F**, processed for OXTR binding; scale bars align for **A**-**B** and **F**-**G**. **C**, **H** Overview DAPI scans of the sections imaged for fluorescence in situ hybridization, showing the locations of the 20X images taken in panels **D**-**E** and **I**-**J**. **D**, **I**. Representative 20X images of the duplex fluorescence in situ hybridization results from the NBM. **E**, **J** Representative 20X images of the duplex fluorescence *in situ* hybridization results from the VP; scale bar in panel J applies to all of the 20X fluorescence images, which were all equally processed for *ChAT* (red) and *OXTR* (green) in situ hybridization

**Fig. 5:**
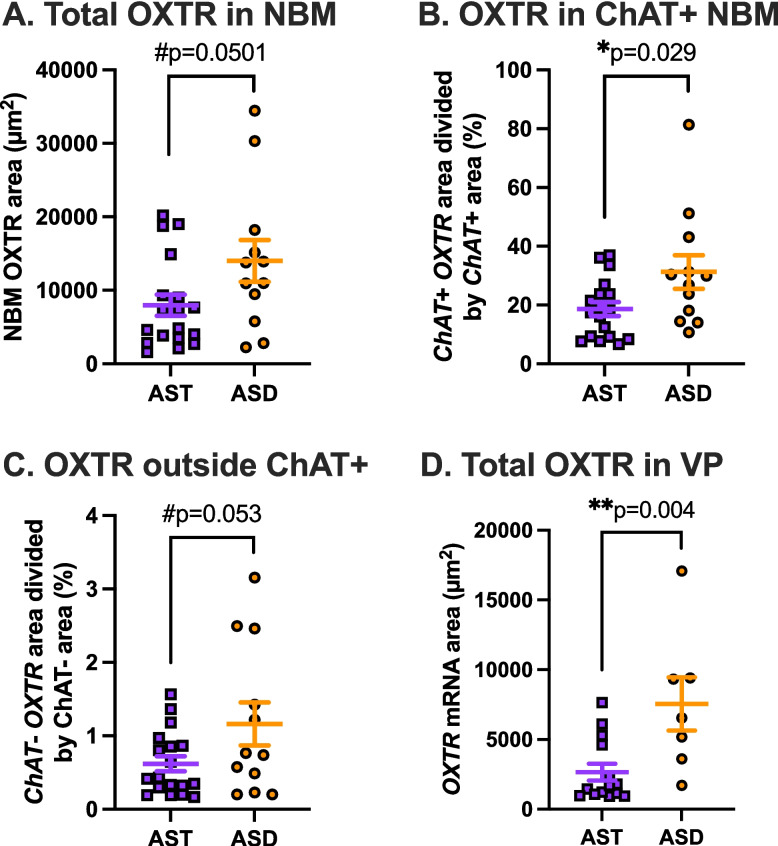
*OXTR* gene expression metrics in the human basal forebrain across groups. **A** Total *OXTR* mRNA area in the nucleus basalis of Meynert (NBM) neared statistical significance; N_AST_ = 18, N_ASD_ = 12, *p =* 0.0501. **B** *OXTR* mRNA area within the cholinergic areas of the NBM was significantly greater in specimens from donors with autism (ASD, orange circles) than allistic donors (AST, purple squares); N_AST_ = 18, N_ASD_ = 12, *p =* 0.029. **C** *OXTR* mRNA area outside of the cholinergic areas of the NBM neared statistical significance; N_AST_ = 18, N_ASD_ = 12, *p =* 0.0530. **D** *OXTR* mRNA area in the ventral pallidum (VP) was significantly greater in ASD specimens than AST specimens. **B** *OXTR* mRNA area in the ventral pallidum (VP) was significantly greater in ASD specimens than allistic controls; N_AST_ = 14, N_ASD_ = 7, *p =* 0.004. Error bars represent ± SEM. #*p* < 0.10, *p < 0.05, **p < 0.01. For all four plots in this figure, group differences were statistically evaluated by regressing the *OXTR* mRNA metric in question (y-axis) on neurotype (x-axis)

Only two *OXTR* mRNA metrics exhibited significant differences between neurotypes: *ChAT*+ *OXTR* area (*p =* 0.029) and VP *OXTR* area (*p =* 0.004). For both *ChAT*+ *OXTR* area and VP *OXTR* area, our estimated marginal mean values were higher for ASD than AST (Table 1; Figs. 4 and 5). For our other two *OXTR* mRNA metrics (total NBM *OXTR* area and *ChAT- OXTR* area), the neurotype group differences were *p =* 0.0501 and *p =* 0.0530, respectively, verging at the level of significance.

**Table 1:** Estimated marginal means and confidence intervals for *OXTR* mRNA metrics

Region	Metric	Neurotype	Mean	Standard error	95% Confidence interval lower bound	95% Confidence interval upper bound
NBM	OXTR	Autistic	12409	2351	8061	17691
NBM	OXTR	Allistic	6972	1439	4337	10231
NBM	ChAT + OXTR	Autistic	31.3	4.23	22.6	39.9
NBM	ChAT + OXTR	Allistic	18.7	3.45	11.6	25.7
NBM	ChAT- OXTR	Autistic	1.163	0.208	0.736	1.589
NBM	ChAT- OXTR	Allistic	0.619	0.17	0.271	0.968
VP	OXTR	Autistic	6831	1446	4139	10193
VP	OXTR	Allistic	2292	592	1220	3699

### *OXTR* mRNA association with OXTR binding density from autoradiography

**Fig. 6:**
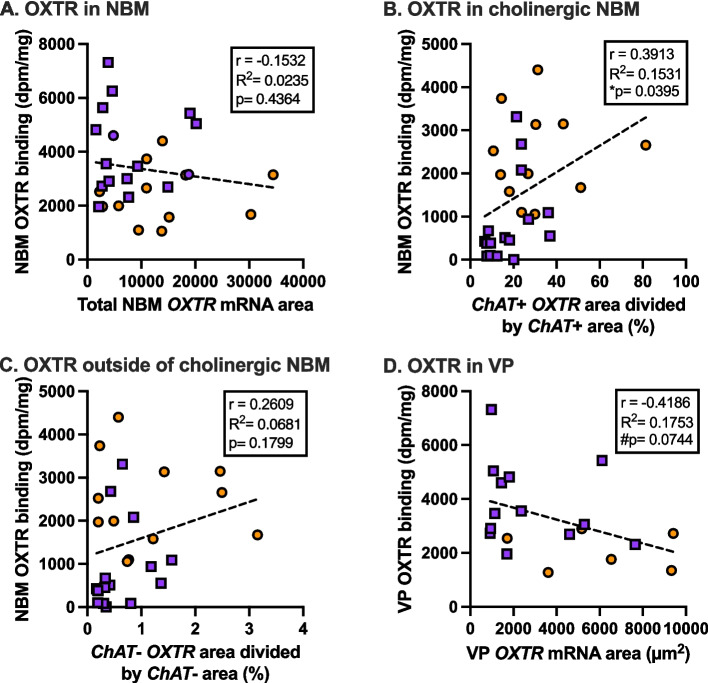
Correlations between *OXTR* mRNA and OXTR binding across specimens. **A** There is no association across all specimens between *OXTR* mRNA and receptor binding levels in images across the nucleus basalis of Meynert (NBM), N_all_ = 26. **B** There is a significant positive correlation between *OXTR* gene expression and receptor binding levels in the cholinergic neurons of the NBM, N_all_ = 26. **C** There is no association between *OXTR* mRNA and receptor binding levels in the NBM outside of the cholinergic neurons, N_all_ = 26. **D** There is a trending negative association between *OXTR* mRNA and receptor binding levels in ventral pallidum (VP), N_all_ = 18. Pearson’s correlation was used in all cases. ASD, orange circles; AST, purple squares

In the NBM, there was a significant positive association between *ChAT*+ *OXTR* area and OXTR binding across all specimens (*r* = 0.3913, *p =* 0.0395; Fig. 6B). There were no significant associations between VP *OXTR* area and OXTR binding density, although there was a trend toward an overall negative association between VP *OXTR* area and OXTR binding density across all samples (*r* = −0.4186, *p =* 0.0744; Fig. 6D). Neither of the other two NBM *OXTR* metrics (total area and *ChAT- OXTR*) were significantly associated with OXTR binding density.

### OXTR binding density association with *OXTR* SNPs

**Fig. 7:**
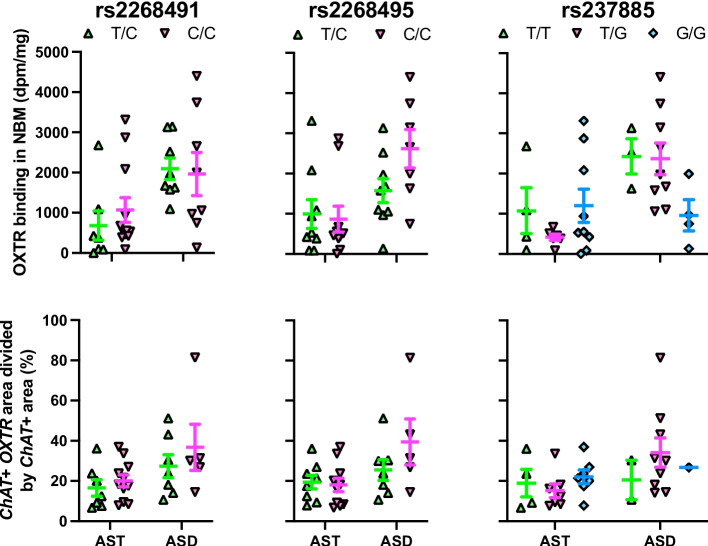
Lack of relationship between genotype and OXTR measures in the nucleus basalis of Meynert (NBM). Top row of graphs shows OXTR binding levels by genotype in the NBM for the three SNPs of interest. The ASD samples initially showed a trend toward a significant effect of genotype for SNP rs2268495, but the result did not survive correction for multiple comparisons. The ASD samples also initially showed a significant effect of genotype for SNP rs237885, which also did not survive correction. Bottom row of graphs show *OXTR* mRNA levels within *ChAT* + areas by genotype for the three SNPs of interest. There were no significant associations between genotype and *ChAT* + *OXTR* mRNA for any of the SNPs. Error bars represent ± SEM

**Fig. 8:**
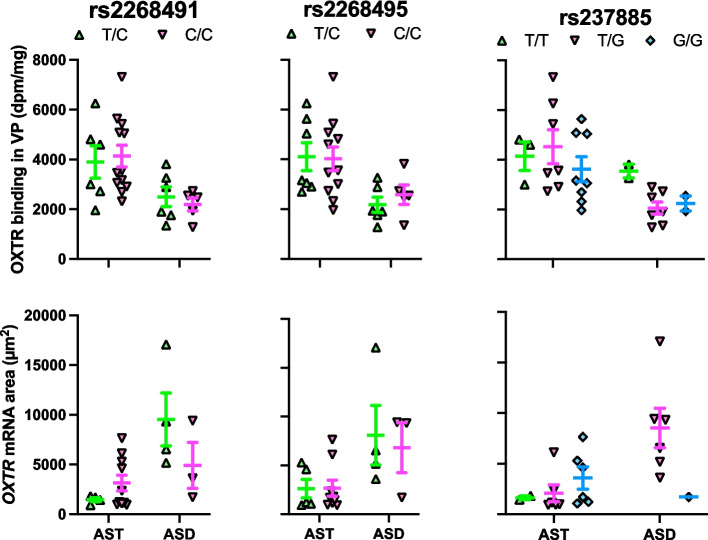
Lack of relationship between genotype and OXTR measures in the ventral pallidum (VP). Top row of graphs show genotype by OXTR binding levels in the VP for the three SNPs of interest. The ASD samples had a trend toward an effect of genotype on OXTR binding levels for rs237885, which did not survive statistical correction. There were no significant associations between genotype and *OXTR* mRNA in the VP for any of the SNPs. Error bars represent ± SEM

Two of our models yielded associations worth noting. For the NBM OXTR binding density (Fig. 7) with only ASD samples (*R*^2^ = 0.33), rs2268495 had a p-value of 0.057 (adjusted *p*-value = 0.228), and rs237885 had a *p*-value of 0.028 (adjusted *p*-value = 0.224). For the VP OXTR binding density (Fig. 8) with ASD only (*R*^2^ = 0.90), rs237885 had a p-value of 0.092 (adjusted *p*-value = 0.658). But none of the correlations survived correction for multiple comparisons, as seen in the adjusted p-values reported here.

### *OXTR* mRNA association with *OXTR* SNPs

No significant correlations between our SNPs of interest and *OXTR* mRNA were found in either the NBM (Fig. 7) or VP (Fig. 8).

### *OXTR* mRNA correlation with age

**Fig. 9:**
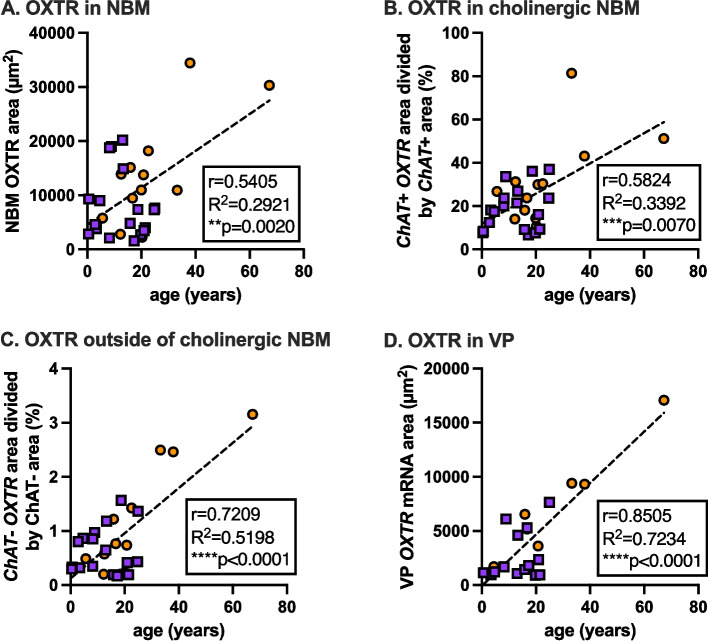
*OXTR* gene expression increases with specimen age, especially in autism. In all four of our *OXTR* mRNA metrics, we found significant positive associations between *OXTR* mRNA levels and donor age. Across all four measures, this is being driven by ASD specimens, and the associations remain significant for all measures except total NBM *OXTR* area when the oldest two ASD specimens are removed. Pearson’s correlation was used in all cases. **A**-**C** N_ASD_ = 12, N_AST_ = 18, N_all_ = 30. **D** N_ASD_ = 7, N_AST_ = 14, N_all_ = 21. ASD, orange circles; AST, purple squares

*OXTR* mRNA was significantly positively correlated with age for all four of our *OXTR* mRNA metrics (Fig. 9). Our Pearson’s correlations for age with total NBM *OXTR* area, *ChAT*+ *OXTR* area, *ChAT- OXTR* area, and VP *OXTR* area were *r* = 0.5405 (*p =* 0.0020), *r* = 0.5824 (*p =* 0.0070), *r* = 0.7209 (*p* < 0.0001), and *r* = 8505 (*p* < 0.0001), respectively. It initially appeared that these strong positive correlations might have been driven by a single 67-year old sample, but excluding that possible outlier from the analysis resulted in three of the four outcomes remaining significant (all three at *p* < 0.01), except total NBM *OXTR* area, which became trending at *p =* 0.0684. We also re-ran the correlations without the oldest two specimens (67-year old and 38-year old), and the three significant positive associations remained (*ChAT*+ *OXTR* area at p0.0107, *ChAT- OXTR* area at *p =* 0.0412, and VP *OXTR* area at *p =* 0.0121), with total NBM *OXTR* area no longer significant or trending (*p =* 0.7547).

## Discussion

This study sought to evaluate whether there are differences in *OXTR* gene expression or genotype between ASD and AST samples in two regions of interest in the human basal forebrain that were previously reported to have differential levels of OXTR protein, as measured by receptor binding density. We found increased *OXTR* mRNA in the cholinergic *ChAT*+ NBM and the VP of ASD specimens. The NBM result aligns with our previous finding of higher OXTR binding in the NBM in ASD, and we also found that the only significant positive correlation between OXTR binding density and our *OXTR* mRNA metrics in the NBM was the *ChAT*+ *OXTR* (not the total NBM *OXTR* or *ChAT- OXTR*). These results, as well as the strong colocalization of OXTR on *ChAT* neurons, point toward a functionally relevant circuit involving OXT’s ability to modulate the cholinergic system of the human brain. It should be noted that the primary purpose of our study was to align previously collected OXTR binding data (at a macroscopic scale) with *OXTR* gene expression (at the microscopic scale). Thus, one limitation of this study is the lack of systematic, high-resolution quantification at the cellular level, namely 60× magnification. Future studies into intracellular *OXTR* mRNA and OXTR protein localization using high-resolution imaging can provide valuable insights into the molecular etiology of the differences observed in this study and strengthen our understanding of OXT’s relationship with the cholinergic system.

The VP result is counter to our initial hypothesis that the gene expression levels would match what we previously reported: ASD specimens had lower OXTR binding density in the VP. We had previously interpreted the lower levels of OXTR binding in the VP in the context of reduced social reward or reduced social motivation, given the VP’s role in the mesolimbic dopaminergic reward pathway. But in the current study, we found increased *OXTR* mRNA in the VP in ASD, as well as a lack of association between *OXTR* mRNA and receptor binding in the VP. Taken together this result could indicate dysregulation in the translation of *OXTR* mRNA into functional, mature cell-surface protein in the VP in ASD; however, we found a lack of association between OXTR mRNA and binding in the VP across all specimens, which means there could be a similar phenomenon happening in the allistic brain. Although this result was somewhat unexpected, a lack of association between *OXTR* mRNA and binding has recently been reported in the prairie vole brain using the same techniques as the current study [39]. While gene expression and protein levels would be expected to co-vary, this discrepancy can be explained by the ability of mRNA in neurons to be transported to axons [40, 41], which has been observed in *OXTR* mRNA in rodent hypothalamus [42]. In fact, these differences in relative levels of *OXTR* mRNA vs receptor binding across multiple studies and species highlights the importance of using both receptor binding and measurements of *OXTR* gene expression in order to draw strong inferences about the function of OXT in the brain. Future studies are clearly needed to address cellular and molecular characteristics of the OXT/OXTR signaling pathway in a region-by-region approach to assess whether differences in gene expression, protein translation, or receptor internalization are contributing to ASD neuropathology and symptom expression.

Both *OXTR* mRNA within *ChAT*+ areas in the NBM and *OXTR* mRNA within the VP showed significant differences by neurotype, while NBM *ChAT- OXTR* mRNA and total *OXTR* mRNA in the NBM both trended towards significance. The fact that the *OXTR* mRNA levels outside of the *ChAT*+ areas were only trending toward significant differences between groups points toward functional consequences of increased *OXTR* gene expression specifically within the cholinergic neurons of the NBM in ASD, rather across the basal forebrain more broadly. This result of increased *ChAT*+ *OXTR* mRNA levels aligns with our findings and interpretation from our previous study: it is possible that increased *OXTR* gene expression and increased OXTR binding in this area of the brain in ASD underlies an increased sensitivity of the cholinergic basal forebrain to OXT release, which could in turn be related to difficulties with social visual attention, because the basal forebrain’s cholinergic input to the neocortex mediates selective and sustained attention to visual stimuli [43]. Interestingly, when we investigated whether *OXTR* mRNA levels in *ChAT*+ areas were correlated with OXTR binding densities measured in anatomically adjacent sections from the same specimens, there was a significant positive association between *OXTR* mRNA and OXTR binding, but only for *ChAT*+ *OXTR* levels, not for any other NBM *OXTR* measure. Thus, it appears that as *OXTR* gene expression increases in the cholinergic magnocellular neurons of the human NBM, so does the mature cell surface OXTR receptor protein. We interpret this collection of results to imply overall elevated OXTR production in ASD in a functionally important collection of neurons that influences the levels of functional cell surface OXTRs. Given that *OXTR* mRNA was not significantly positively correlated with OXTR binding density in any of our other areas, it is possible that imbalances in gene expression levels for molecules involved in OXT secretion (CD38) and breakdown (LNPEP), which have recently been reported in ASD [44], may be involved in dysregulation in the OXT system, from both the peptide and receptor sides.

There were no effects of genotype at any of the three SNPs on *OXTR* gene expression or OXTR binding levels in either of our two regions of interest. Each of the three SNPs that we investigated (rs2268491, rs2268495, rs237885) are intronic and have been associated with ASD across studies, and two of these (rs2268491, rs237885) emerged as significant in the largest meta-analysis of *OXTR* and ASD to date [10]. So our lack of any significant findings here is likely due to sample size. Although our study is the first to our knowledge to attempt to link *OXTR* genotype directly to neural OXTR phenotypes in tissue sections with preserved anatomy, it is likely statistically underpowered to detect differences. Additionally, because the specific SNP BeadArray we used only allowed us to assess a priori SNPs, the other known *OXTR* SNPs that have been associated with ASD were not measured.

We found significant positive correlations between all four of our *OXTR* mRNA metrics and age across all specimens. Interestingly, in our previous binding study, we only found a significant association between OXTR binding levels and age in the VP (not NBM), and the direction of the association was opposite: OXTR binding was highest in early childhood and was negatively associated with increasing age across all specimens. In our current study, it appeared that these significant correlations between age and *OXTR* mRNA could be being driven by one specimen from a 67-year-old donor with ASD; but when this data point is removed, the significant positive associations remain (total NBM *OXTR* mRNA becomes trending). Even when the next oldest 38-year-old donor is removed from the analysis, those three remaining significant associations still remain. Thus, it appears that while our previous study indicated that OXTR binding may be reduced in the VP in ASD in early childhood and adolescence, the current study suggests that *OXTR* gene expression levels may be elevated across these two regions with increasing age, although future studies are needed to confirm this result, given the limited number of specimens older than 30 included in our study.

Given the postmortem nature of this study, our sample size is lower than would be expected for similar studies of living subjects. However, compared to most histological studies of the postmortem brain of humans and nonhuman primates, our sample size is double to triple what is typical. Due to unavailability of clinical data associated with our specimens, we were unable to evaluate whether symptom severity was associated with any of our outcome measures.

## Conclusions

The group differences observed in this study provide a more nuanced picture of the ways in which the OXT system interacts with ASD and opens the door to future studies examining post-translational transport, modifications and degradation of OXTR. Beyond the assessment of neurotype group differences, the resulting colocalization of *OXTR* mRNA in the cholinergic neurons in the human basal forebrain is striking. To our knowledge this is the first demonstration of *OXTR* mRNA in the cholinergic neurons of the human basal forebrain, a result that would not have been possible through the use the transcriptomic methods used in brain tissue homogenates. This result provides a critical piece of anatomical information to the “puzzle” of understanding OXT’s function in the human brain. By showing that the cholinergic magnocellular neurons of the human basal forebrain are expressing high levels of *OXTR* mRNA, our results provide a mechanism by which OXT released in the basal forebrain can directly bind to and modulate the function of the neurons that provide cholinergic input to the neocortex. This critical anatomical piece of evidence supports the idea that OXT is likely acting in the human brain as a “modulator of modulators”—an idea that has been increasing in popularity over the last few years [45–48]. As differences in social attention can contribute to more substantial differences in social perception and social cognition, future studies should assess the interaction between OXT and the cholinergic system in the modulation of behavior, similar to how studies of OXT’s interplay with dopamine [49] and with the opioid system [50, 51] are now contributing to a more complex understanding of the mechanisms by which OXT can impact physiology and behavior.

## Supplementary Information

Supplementary Material 1.

## Data Availability

The datasets used and/or analyzed during the current study are available from the corresponding author on reasonable request.

## Abbreviations

ASD: Autism Spectrum Disorder
AST: Allistic
ChAT: Choline Acetyltransferase
fISH: Fluorescence In Situ Hybridization
GSA: Illumina Infinium Global Screening Array
IN-OXT: Intranasal Oxytocin
IRB: Institutional Review Board
NBM: Nucleus Basalis of Meynert
OXT: Oxytocin
OXTR: Oxytocin Receptor
RT: Room Temperature
SNP: Single-Nucleotide Polymorphism
USU: Utah State University
VP: Ventral Pallidum
