# Supplementary material for: Oxytocin receptor gene expression in the basal forebrain in autism: association with receptor binding levels and single nucleotide polymorphisms
Source: J Neurodev Disord. 2026 Feb 19;18:16. doi: 10.1186/s11689-026-09678-0 (PMC13019852; doi:10.1186/s11689-026-09678-0)
Supplement: Supplementary file 1 — Supplementary Material 1. [file 11689_2026_9678_MOESM1_ESM.docx]

**Positive and negative controls**

**
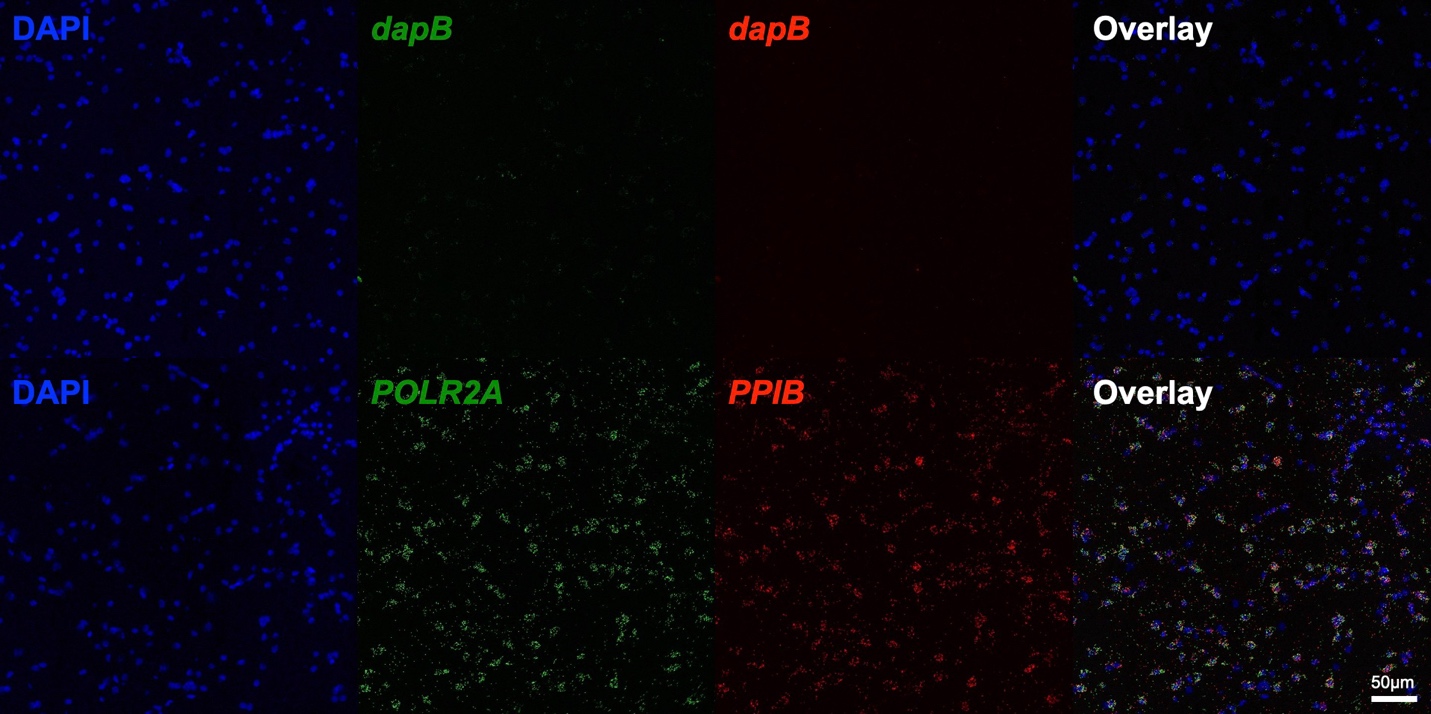
**

**Supplementary Figure 1. Representative images from the positive and negative control probes**. Top row: 20X images from the duplex negative control probe (bacterial gene *dapB* in both channels). Bottom row: 20X images from the duplex positive control probe (mammalian housekeeping gene *POLR1A* in channel 1 and *PPIB* in channel 2). All images shown were taken from the same specimen.

**Results using signal intensity (brightness)**

*OXTR mRNA Comparison Between Neurotypes*

Only two *OXTR* mRNA metrics exhibited differences between neurotypes: *ChAT+ OXTR* (p = 0.052) and log-transformed VP *OXTR* (p = 0.003). For both *ChAT+ OXTR* and VP *OXTR*, our estimated marginal mean values were higher for ASD than AST (Supplementary Table 1; Supplementary Figure 2), with VP *OXTR* reaching statistical significance, but *ChAT+ OXTR* trending very near to significance.

Supplementary Table 1: Estimated marginal means and confidence intervals for *OXTR* mRNA metrics

| Region | Neurotype | Mean | Standard Error | 95% Confidence Interval Lower Bound | 95% Confidence Interval Upper Bound |
| --- | --- | --- | --- | --- | --- |
| NBM | Autistic | 26.6 | 3.21 | 20 | 33.2 |
|  | Allistic | 18.2 | 2.62 | 12.8 | 23.5 |
| VP | Autistic | 398486 | 127064 | 204441 | 776711 |
|  | Allistic | 105045 | 23685 | 65527 | 168394 |

*Metrics are ChAT+ OXTR and VP OXTR for NBM and VP respectively.*

**Supplementary Figure 2. OXTR gene expression in the human basal forebrain across groups**. A. OXTR mRNA signal brightness within the cholinergic areas of the nucleus basalis of Meynert (NBM) was significantly greater in specimens from donors with autism (ASD, orange circles) than allistic donors (AST, purple squares). N_AST_=18, N_ASD_=12, p=0.052 B. OXTR mRNA signal brightness (log transformed for normality) in the ventral pallidum (VP) was significantly greater in ASD specimens than allistic controls. N_AST_=14, N_ASD_=7, p=0.003. Error bars represent ± SEM. * p<0.05, ** p<0.01. For both plots in this figure, group differences were statistically evaluated by regressing the OXTR mRNA metric in question (y-axis) on neurotype (x-axis).

*OXTR mRNA Association with OXTR Autoradiography*

In the NBM, there was a significant positive association between *ChAT+ OXTR* and OXTR binding across all specimens (slope=62.81, R^2^=0.285, p=0.003). *ChAT+ OXTR* was much more positively associated with OXTR binding for AST (slope=81.91, R^2^=0.445, p=0.005) than *ChAT+ OXTR* in ASD, which was not significantly associated with OXTR binding (slope=3.964, R^2^=0.0018, p=0.895) (Supplementary Figure 3). There were no significant associations between VP *OXTR* and OXTR binding density, although there was a trend toward an overall negative association between VP *OXTR* and OXTR binding density across all samples (slope=-686.3, R^2^=0.1970, p=0.057).

**Supplementary Figure 3. Correlations between OXTR mRNA and OXTR binding across specimens.** A. There is a significant positive association across all specimens between OXTR gene expression and receptor binding levels in the nucleus basalis of Meynert (NBM), which appears to be driven by a significant positive association in the allistic (AST) samples only (purple squares). Autistic (ASD) specimens (orange circles) show no correlation between OXTR mRNA and binding. N_ASD_=11, N_AST_=15, N_all_=26. B. There were no significant correlations between OXTR gene expression and receptor binding levels in the ventral pallidum (VP) in either AST or ASD specimens, although there was a trending negative association across all specimens. N_ASD_=6, N_AST_=12, N_all_=18. Pearson’s correlation was used in all cases.

*OXTR Binding Density Association with OXTR SNPs*

Two of our models yielded associations worth noting. For the NBM OXTR (Figure 6) with only ASD samples (R^2^=0.33), rs2268495 had a p-value of 0.057 (adjusted p-value=0.228), and rs237885 had a p-value of 0.028 (adjusted p-value=0.224). For the VP OXTR (Figure 7) with ASD only (R^2^=0.90), rs237885 had a p-value of 0.092 (adjusted p-value=0.658). But none of the correlations survived correction for multiple comparisons, as seen in the adjusted p-values reported above.

**Supplementary Figure 4. Lack of relationship between genotype and OXTR measures in the nucleus basalis of Meynert (NBM).** Top row of graphs shows OXTR binding levels by genotype in the NBM for the three SNPs of interest. The ASD samples initially showed a trend toward a significant effect of genotype for SNP rs2268495, but the result did not survive correction for multiple comparisons. The ASD samples also initially showed a significant effect of genotype for SNP rs237885, which also did not survive correction. Bottom row of graphs show *OXTR* mRNA levels within *ChAT*+ areas by genotype for the three SNPs of interest. There were no significant associations between genotype and *ChAT+ OXTR* mRNA for any of the SNPs. Error bars represent ± SEM.

**

**Supplementary Figure 5. Lack of relationship between genotype and OXTR measures in the ventral pallidum (VP).** Top row of graphs show genotype by OXTR binding levels in the VP for the three SNPs of interest. The ASD samples had a trend toward an effect of genotype on OXTR binding levels for rs237885, which did not survive statistical correction. There were no significant associations between genotype and *OXTR* mRNA in the VP for any of the SNPs. Error bars represent ± SEM.

*OXTR* *mRNA Association with OXTR SNPs*

No significant correlations between our SNPs of interest and *OXTR* mRNA were found in either the NBM (Supplementary Figure 4) or VP (Supplementary Figure 5).

*OXTR mRNA Correlation with Age*

*OXTR* mRNA was significantly positively correlated with age for both the NBM and VP (Supplementary Figure 6). Our Pearson’s correlation for *ChAT+ OXTR* mRNA with age was r=0.402 with significance p=0.028, and for VP *OXTR* mRNA was r=0.813 with significance p=0.004. Correlation by diagnosis in the NBM (Figure 8A) resulted in a trending correlation for *ChAT+ OXTR* mRNA with age (r=0.522; p=0.082) for ASD only, and no significant correlation for AST (r=0.035; p=0.900). For the VP (Figure 8B), correlation by diagnosis revealed a significant association for ASD (r=0.961; p<0.01), and no significant correlation for AST (r=0.214; p=0.463).

**Supplementary Figure 6. OXTR gene expression increases with specimen age, especially in autism.** In both of our regions of interest, we found a significant positive association between OXTR mRNA levels and donor age. In both regions, this appears to be driven by ASD specimens, which showed a significant positive association in the VP, but only a trending association in NBM. Pearson’s correlations were performed for each grouping in both plots. A. N_ASD_=12, N_AST_=18, N_all_=30. B. N_ASD_=7, N_AST_=14, N_all_=21.

**Effect of batch**

*Background*

Our samples were initially grouped into three different assays (referred to as Week 1, Week 2, and Week 3). Once imaging was complete, the samples were analyzed for batch-wise differences in brightness. Since upon initial analysis Week 3 had significantly lower brightness across all channels, that set of samples was re-run using backup slides (referred to as Week 3 RR). Appendix S1 compares Week 1, Week 2 and Week 3 RR, the three sets which were used for this study.

*Capture Delta Statistics*

Capture delta is here defined as the time in days between completion of the assay and imaging of a specimen in said assay. Delta values were obtained for all specimens with NBM images (N = 32). The NBM was selected because the histology was fairly consistent between specimens, and the images were guaranteed to include all channels. Positive controls could not be used because of inconsistent histology and sampling. The mean capture delta was 31.8 and the median capture delta was 30, with a range of [20, 52]. Plotted in a histogram, delta values appear to have a unimodal right-skewed distribution, which could interfere with the interpretation of some results here (See Supplementary Figure 7).

One-way ANOVA was performed on Capture Delta by Assay Week, and a p-value of << 0.01 was obtained. Tukey’s HSD was used to obtain pair-wise p-values, and significant differences were found between *Week 1 & Week 2* (p << 0.01), and *Week 2 & Week 3* (p < 0.01). Weeks *1 & 3* were not found to differ significantly. See Supplementary Figure 8 for a boxplot comparing the same variables.

*Image Brightness Statistics*

We used four measures of image brightness: 1) All channels, 2) DAPI only, 3) OXTR only, and 4) ChAT only. One-way ANOVA was performed on each measure of image brightness, followed by Tukey’s HSD for pair-wise comparisons (See Supplementary Table 2). A boxplot was also created to graphically compare image brightness across all four measures (See Supplementary Figure 9). In both cases, no significant inter-group differences were observed.

**Analysis of Capture Delta Effects on Image Brightness**

We analyzed potential effects of differential capture deltas on specimen brightness across all four measures. Brightness values for all four measures were plotted and OLS trendlines were overlaid (See Supplementary Figure 10). Although at first glance, there appears to be a consistent negative correlation between Capture Delta and brightness values, the histogram of Brightness across all channels (rescaled by minimum brightness in range) displays an apparent bimodal distribution (See Supplementary Figure 11). This bimodal distribution is confirmed by a scatterplot of brightness values for all four measures overlaid with LOESS trendlines and colored by week (See Supplementary Figure 12). The LOESS trendline and assay-wise coloring shows that the negative slope of the trendline is most likely due to outliers in Week 1 than to capture deltas. Univariate OLS regression was then performed for both Brightness by Capture Delta and Brightness by Assay Week across all four measures (See Supplementary Table 3). In all cases, p-values for regression of Brightness on Capture Delta were insignificant with p >> α = 0.05, and regression of Brightness on Assay Week also produced p-values >> α = 0.05.

**Summary of Results**

This analysis showed no significant evidence of differential image brightness due to Capture Delta or Assay Week. This likely means that the issues seen in the initial Week 3 assay results were corrected by re-running the assay. That said, outliers from Week 1 may need to be carefully examined as part of the analysis, since Week 1 brightness appears to have greater variation than the other two weeks.

**Tables**

| **Brightness Measure** | **Overall ANOVA p-value** | **Tukey’s HSD p-value (Week 1-Week 2)** | **Tukey’s HSD p-value (Week 1-Week 3)** | **Tukey’s HSD p-value (Week 2-Week 3)** |
| --- | --- | --- | --- | --- |
| All Channels | 0.98 | 1.00 | 0.98 | 0.99 |
| DAPI | 0.394 | 1.00 | 0.46 | 0.51 |
| OXTR | 0.382 | 0.98 | 0.41 | 0.55 |
| ChAT | 0.90 | 0.89 | 0.98 | 0.95 |

Supplementary Table 2: P-values for ANOVA and Tukey’s HSD (Brightness Measure ~ Assay Week).

| **Brightness Measure** | **p-value for *Brightness ~ Capture Delta*** | **p-value for *Brightness ~ Assay Week*** |
| --- | --- | --- |
| All Channels | 0.29 | Week 2 = 0.95 / Week 3 = 0.84 |
| DAPI | 0.30 | Week 2 = 0.96 / Week 3 = 0.23 |
| OXTR | 0.49 | Week 2 = 0.85 / Week 3 = 0.21 |
| ChAT | 0.17 | Week 2 = 0.66 / Week 3 = 0.87 |

Supplementary Table 3: P-values for OLS regression models of brightness on either capture delta or assay week.

**Figures**


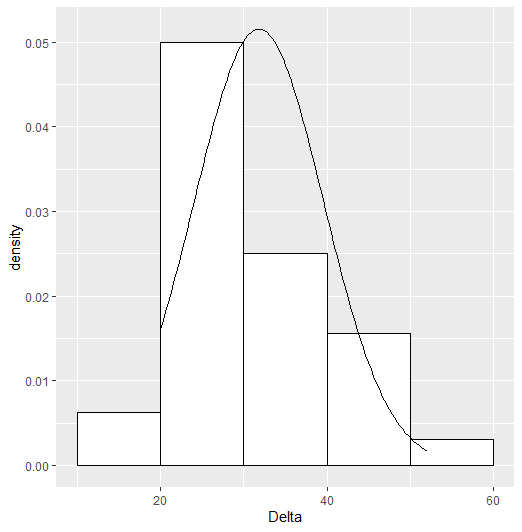


Supplementary Figure 7: Histogram of Capture Delta values.


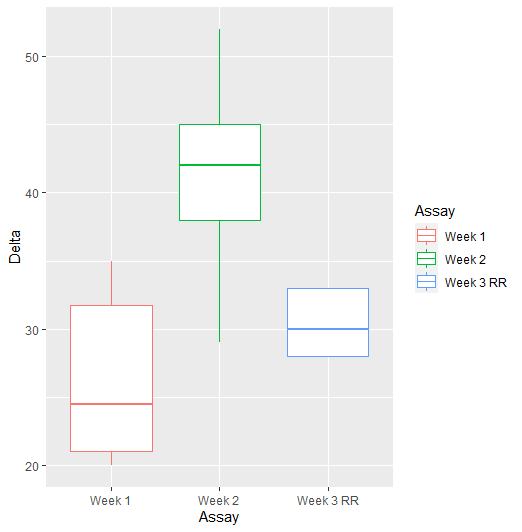


Supplementary Figure 8: Boxplot of Capture Delta by Assay Week.


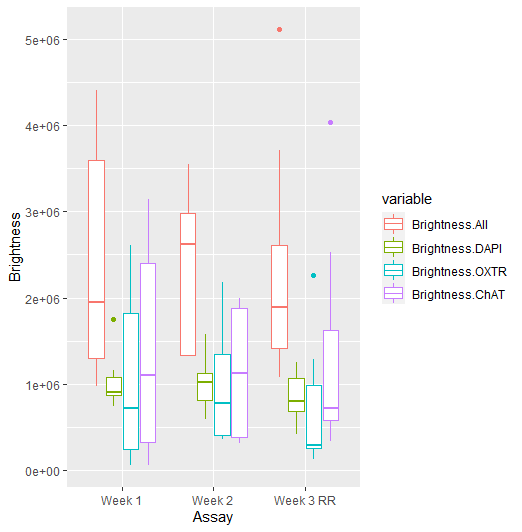


Supplementary Figure 9: Boxplot of Image Brightness measures by Assay Week.


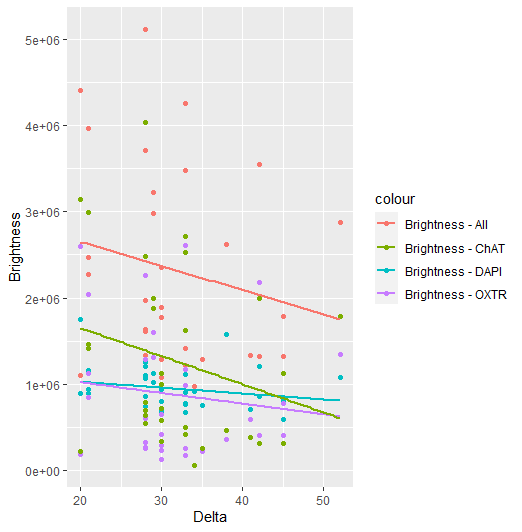


Supplementary Figure 10: Scatterplot of Image Brightness with OLS trendline overlays.


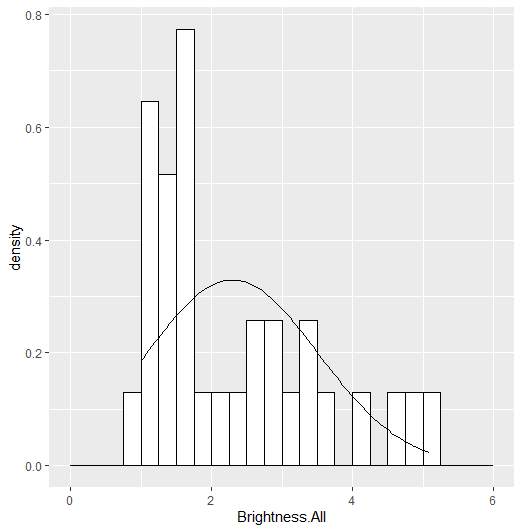


Supplementary Figure 11: Histogram of Brightness across all channels values.


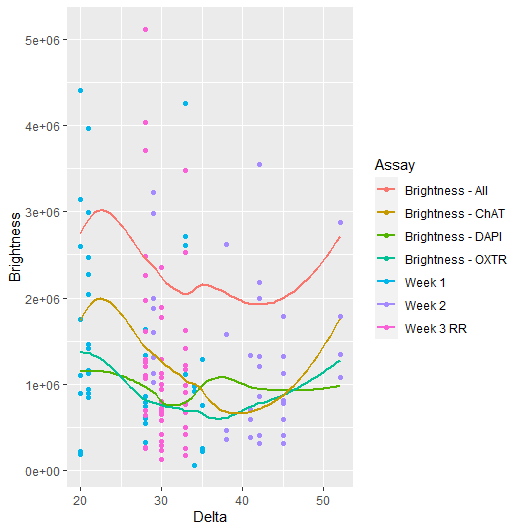


Supplementary Figure 12: Scatterplot of Image Brightness measures, colored by Assay Week, with LOESS trendlines overlaid.
